# Irreducible Anterior Shoulder Dislocation Caused by Soft Tissue Interposition: A Comprehensive Review

**DOI:** 10.7759/cureus.100246

**Published:** 2025-12-28

**Authors:** Ahmed Mohamed, Nabil Elmaleh, Usman Fuad, Adham Elsayed, Ahmed Genena, Marwa Harb, Jumanah Darwish, Ahmed Nagi

**Affiliations:** 1 Trauma and Orthopaedics, Royal Cornwall Hospital, Truro, GBR; 2 Trauma and Orthopaedics, University Hospitals of Morecambe Bay, Lancaster, GBR; 3 Trauma and Orthopaedics, James Paget University Hospital, Great Yarmouth, GBR; 4 Trauma and Orthopaedics, Faculty of Medicine, Helwan University, Helwan, EGY; 5 General Practice, University Hospitals of Derby and Burton NHS Foundation Trust, Derby, GBR; 6 Trauma and Orthopaedics, School of Medicine and Population Health, The University of Sheffield, Sheffield, GBR; 7 Trauma and Orthopaedics, Wirral University Teaching Hospital NHS Foundation Trust, Wirral, GBR; 8 Trauma and Orthopaedics, University Hospitals of Derby and Burton NHS Foundation Trust, Derby, GBR

**Keywords:** anterior dislocation, biceps tendon, glenohumeral joint, irreducible shoulder dislocation, soft tissue interposition, subscapularis

## Abstract

Anterior shoulder dislocation is the most common type of joint dislocation encountered in clinical practice, with the vast majority being successfully managed using closed reduction techniques. However, in rare instances, anatomical obstacles prevent successful closed reduction, necessitating open surgical intervention. Irreducible anterior shoulder dislocations caused by soft tissue interposition represent a challenging clinical entity that requires prompt recognition and appropriate management. This comprehensive review examines the pathophysiology, clinical presentation, diagnostic approaches, and treatment strategies for irreducible anterior shoulder dislocations secondary to soft tissue interposition, with an emphasis on recent literature from 2008 to 2025. The most commonly interposed structures include the long head of the biceps tendon (LHBT), the subscapularis tendon, other rotator cuff components, and, rarely, peripheral nerves. Understanding the mechanisms of irreducibility, recognizing clinical red flags, and implementing appropriate imaging protocols are essential for optimal patient outcomes. The deltopectoral approach remains the standard surgical access, with external rotation maneuvers facilitating the delivery of interposed subscapularis tissue. MRI provides superior soft tissue characterization compared to CT when plain radiographs fail to demonstrate obvious bony pathology. Outcomes are generally favorable when early recognition is coupled with appropriate surgical intervention and structured rehabilitation, although complications, including nerve injury and residual functional deficits, can occur.

## Introduction and background

The glenohumeral joint is the most frequently dislocated joint in the human body, with anterior dislocations accounting for approximately 95%-96% of all shoulder dislocations [1]. The annual incidence of first-time anterior shoulder dislocation ranges from 8 to 23.9 per 100,000 person-years, with a marked predominance in young and active males [2]. While the majority of anterior shoulder dislocations are amenable to closed reduction with success rates of 70%-90%, a small number encounter mechanical obstruction requiring further interventions [3].

Irreducible anterior shoulder dislocation is a rare but clinically significant challenge. The failure of closed reduction can result from osseous blocks, such as displaced fracture fragments or severe Hill-Sachs lesions impacting the glenoid rim, or from soft tissue interposition within the glenohumeral joint space [4]. Soft tissue interposition encompasses various anatomical structures, including the long head of the biceps tendon (LHBT), subscapularis tendon, other rotator cuff components, glenoid labrum, joint capsule, and, rarely, peripheral nerves such as the axillary or musculocutaneous nerves [4]. Early recognition and appropriate surgical intervention are critical to minimize complications, such as neurovascular injury, post-traumatic arthritis, chronic instability, and functional impairment [5].

This narrative review provides a comprehensive examination of irreducible anterior shoulder dislocations caused by soft tissue interposition. We systematically reviewed the epidemiology and risk factors for this rare condition, explored the pathophysiological mechanisms by which different soft tissue structures become interposed, discussed clinical presentation features and diagnostic imaging strategies with emphasis on the role of MRI, examined surgical treatment approaches, including the deltopectoral approach and management of specific interposed structures, and analyzed reported outcomes and complications. This review synthesizes findings from case reports, case series, and systematic reviews to provide practical guidance for clinicians encountering this challenging clinical entity.

## Review

Methods

This narrative review synthesizes the current knowledge on irreducible anterior shoulder dislocations caused by soft tissue interposition, with particular emphasis on contemporary management approaches. A comprehensive systematic literature search was performed across PubMed, MEDLINE, Cochrane Library, and Google Scholar databases to identify relevant publications from January 2008 to October 2025. The search strategy employed Medical Subject Headings (MeSH) terms and keywords including "irreducible shoulder dislocation", "anterior glenohumeral dislocation", "soft tissue interposition", "biceps tendon interposition", "subscapularis interposition", "rotator cuff interposition", and "failed closed reduction".

Studies were included if they were peer-reviewed English-language publications involving acute irreducible anterior shoulder dislocations requiring surgical management with clear documentation of the soft tissue structures causing irreducibility. Studies were excluded if they involved chronic dislocations (more than six weeks after injury), purely osseous causes of irreducibility, or dislocations with ipsilateral humeral shaft fractures. The literature search revealed that most published literature consists of single case reports or small case series, reflecting the rarity of this condition. The most commonly interposed structure was the LHBT, either alone or in combination with greater tuberosity fractures, followed by subscapularis tendon interposition. Table 1 summarizes the key studies identified between 2008 and 2025.

**Table 1:** Summary of key studies on irreducible anterior shoulder dislocations with soft tissue interposition (2008-2025) LHBT: long head of the biceps tendon, MRI: magnetic resonance imaging, CT: computed tomography, ORIF: open reduction and internal fixation.

Study	Year	Study type	Cases	Interposed structure	Associated injury	Preoperative imaging	Method of reduction	Additional procedure	Key findings
Connolly et al. [6]	2008	Case report	1	Subscapularis + LHBT	Supraspinatus, infraspinatus tears	MRI	Arthroscopy converted to open	Subscapularis repair, biceps tenodesis	MRI essential for diagnosis; arthroscopy converted due to joint space occupation
Day et al. [7]	2010	Case report	1	LHBT	None reported	CT	Open deltopectoral	Biceps tenotomy	CT visualization of posteriorly dislocated biceps; multiple reduction attempts led to anterior-posterior-anterior dislocation
Gudena et al. [8]	2011	Case report	1	Musculocutaneous nerve + LHBT	Irreparable massive tears of supraspinatus, infraspinatus, teres minor	Operative findings	Open deltopectoral	Biceps tenodesis, nerve release	Rare nerve interposition; emphasizes caution during reduction attempts
Bayoumy et al. [9]	2017	Technical note	1	Subscapularis + LHBT	None reported	CT and MRI	Open deltopectoral	Subscapularis repair, biceps tenotomy	Detailed surgical technique description; emphasizes delivery of incarcerated subscapularis; no role for arthroscopy
Pantazis et al. [10]	2017	Case report with review	1 (reviewed 30)	LHBT	Greater tuberosity fracture	X-ray, CT	Open deltopectoral	Biceps tenodesis, tuberosity fixation	LHBT most common cause; reviewed 22 reports affecting 30 patients; early surgical intervention mandatory
Ayoubi et al. [11]	2021	Case report	1	Subscapularis	Lesser tuberosity fracture	CT and MRI	Open deltopectoral	ORIF with plate, subscapularis repair	CT essential for surgical planning; prompt recognition critical
Liechti et al. [12]	2022	Systematic review	12	Various (LHBT most common)	Various	Various	11 open, 1 arthroscopic	Various	Low recurrence; no redislocation during follow-up; comprehensive 21st century analysis
Frank et al. [13]	2022	Case report with review	1	Rotator cuff sleeve avulsion	None specified	MRI	Open deltopectoral	Suture bridge repair	Early intervention emphasized; detailed repair technique
Shafieioun et al. [14]	2024	Case report with review	1	LHBT	None specified	MRI	Open reduction	Not specified	Rare posterolateral pattern; MRI critical for diagnosis
Sifi et al. [15]	2025	Case report	1	Subscapularis	Lesser tuberosity fracture	X-ray, CT, MRI	Open deltopectoral	Lesser tuberosity fixation, subscapularis repair	Constant-Murley score 85 at 12 months; orthogonal views essential

The systematic review by Liechti et al. represents the most comprehensive analysis of this condition in the 21st-century literature, identifying 12 cases that required open management. The most common mechanical block was an incarcerated LHBT, followed by the rotator cuff tendons, particularly the subscapularis. Eleven patients were treated with open reduction, and one with arthroscopic reduction. Patients had no recurrent dislocation during follow-up periods ranging from six weeks to two years. Complications included one brachial plexopathy and one musculocutaneous nerve palsy, both demonstrating gradual improvement [12].

Key case reports have highlighted important considerations. Connolly et al. emphasized the role of MRI in diagnosing subscapularis interposition [6]. Bayoumy et al. provided critical technical details, emphasizing that arthroscopy has no role when the joint space is occupied by the subscapularis tendon, and described the use of external rotation to deliver the incarcerated subscapularis [9]. Gudena et al. presented a rare complication of musculocutaneous nerve interposition, emphasizing the need for extreme caution during reduction attempts [8]. Pantazis et al. conducted a comprehensive literature review, revealing 22 reports affecting 30 patients, confirming that LHBT interposition was the most common obstacle [10].

Epidemiology and risk factors

Irreducible anterior shoulder dislocations occur predominantly in male patients in their third to fifth decades of life, accounting for approximately 80%-85% of cases [12]. In older populations, rotator cuff injury is highly suspected, with studies demonstrating rotator cuff involvement in up to 30% of dislocations in patients over 40 years and up to 80% in patients over 60 years [16].

Several risk factors have been identified. High-energy trauma increases the likelihood of complex soft tissue disruption [11]. Associated fractures create additional anatomical derangements that facilitate soft tissue interposition. Delayed presentation allows for progressive muscle spasms and soft tissue edema, which can complicate reduction attempts [17]. Patient-specific factors include pre-existing rotator cuff pathology, shoulder instability, or previous dislocations. Neurological conditions causing seizures represent a unique risk factor, as violent muscular contractions can produce complex dislocation patterns with soft tissue entrapment [18].

Pathophysiology and mechanisms of soft tissue interposition

Understanding the mechanisms by which soft tissue structures become interposed requires an appreciation of the complex three-dimensional anatomy of the glenohumeral joint and the biomechanics of dislocation. During anterior dislocation, the humeral head translates anteriorly and inferiorly, rupturing or stretching the anterior capsulolabral complex. This violent displacement can avulse or tear adjacent soft tissue structures, which may then migrate into the joint space [19].

Long Head of the Biceps Tendon

The LHBT is the most commonly reported structure causing irreducible anterior shoulder dislocations [12]. The tendon's anatomical course makes it particularly vulnerable to interposition. Originating from the supraglenoid tubercle and superior labrum, the LHBT descends through the rotator interval before entering the bicipital groove. During anterior dislocation, the LHBT can be avulsed from its superior attachment or torn mid-substance, allowing the free end to migrate posteriorly and become trapped between the posterolateral aspect of the humeral head and the glenoid [10,20]. The mechanism frequently involves concomitant greater tuberosity fractures [20]. Day et al. demonstrated that the biceps tendon can remain interposed even when the direction of dislocation changes during multiple reduction attempts [7]. Shafieioun et al. described a rare posterolateral pattern of biceps dislocation and incarceration that required MRI for definitive diagnosis [14].

Subscapularis Tendon

The subscapularis is the second most common cause of soft tissue interposition [6,9,11]. As the largest and most powerful rotator cuff muscle, the subscapularis plays a critical role in maintaining anterior shoulder stability. During anterior dislocation, the subscapularis can sustain complete avulsion from its insertion on the lesser tuberosity, full-thickness tears through its substance, or sleeve avulsions [21]. The torn subscapularis tissue can prolapse into the glenohumeral joint space, with a substantial bulk creating a significant mechanical obstacle to reduction [15]. Lesser tuberosity fractures can further complicate the anatomy by creating displaced bony fragments attached to subscapularis tissue, forming a composite block to reduction [11,15].

Other Rotator Cuff Components

While less common, other rotator cuff components, including the supraspinatus, infraspinatus, and teres minor, can become interposed in the setting of massive rotator cuff tears [22]. These scenarios occur in older patients with pre-existing degenerative rotator cuff disease or following high-energy trauma. The torn cuff tissue can herniate into the glenohumeral joint, and the retracted tendon stumps may become incarcerated [10,13,16].

Labrum, Capsule, and Nerve Interposition

The glenoid labrum and joint capsule can also contribute to irreducibility. Large Bankart lesions involving substantial labral tissue can fold into the joint space, creating a soft tissue wedge [19]. The rarest reported cause involves the entrapment of peripheral nerves, particularly the musculocutaneous and axillary nerves [8,23]. Gudena et al. reported musculocutaneous nerve interposition in conjunction with biceps tendon entrapment, emphasizing that this rare complication requires open reduction and careful nerve release [8]. These cases carry a significant risk of permanent neurological deficits and require meticulous surgical techniques.

Clinical presentation and diagnosis

Clinical Features

Patients with irreducible anterior shoulder dislocations present with features such as severe pain, inability to move the affected arm, visible deformity with loss of normal deltoid contour, and the shoulder held in slight abduction and external rotation. However, certain features should raise suspicion for irreducibility [24]. Failed closed reduction attempts under adequate anesthesia and muscle relaxation are the primary indicators. Typically, two to three attempts at closed reduction should be performed before considering the dislocation irreducible [25]. Persistent subluxation or incomplete reduction on post-reduction radiographs should prompt further investigation. Severe pain disproportionate to typical dislocation may indicate nerve entrapment [8].

Neurological and vascular examinations are critical. The axillary nerve is the most commonly affected nerve with anterior shoulder dislocation [26]. While arterial injuries are uncommon in young patients, they can occur in elderly patients, particularly those over 50 years with atherosclerotic disease [27]. Thus, careful assessment of the detailed motor and sensory status of the upper limb nerves and detailed vascular assessment, including radial pulse and capillary refill time, should be clearly documented before and after reduction.

Imaging

Initial imaging begins with standard radiographic views, including anteroposterior, lateral scapular Y-view, and axillary views. Subtle radiographic signs suggesting persistent subluxation include abnormal glenohumeral joint space widening, lack of concentric reduction with a visible gap, persistent overlap on anteroposterior views, and altered relationship on axillary views [28]. However, standard radiographs have limited sensitivity for detecting soft tissue interposition [15].

CT provides excellent visualization of bony structures, allowing accurate assessment of associated fractures [29]. Three-dimensional reconstructions can clarify complex fracture patterns. Day et al. successfully used CT to visualize the posteriorly dislocated biceps tendon [7]. Ayoubi et al. emphasized that CT is essential for surgical planning when fractures are present [11]. While CT excels at demonstrating osseous pathology, its ability to identify soft tissue interposition is limited [30].

MRI represents the gold standard for diagnosing soft tissue interposition. MRI provides superior soft tissue contrast and enables direct visualization of interposed structures [10]. Specific MRI findings include direct visualization of the LHBT displaced posteriorly within the joint, subscapularis tendon avulsion with interposed muscle tissue, rotator cuff tears with cuff tissue herniation, labral displacement, and characteristic patterns of bone marrow edema [31]. Multiple case reports have emphasized that MRI is critical for diagnosing soft tissue interposition patterns [6,9,14,16]. When high-quality plain radiographs fail to show any bony injury in an irreducible dislocation, MRI is the imaging modality of choice [32].

Treatment strategies

Surgical Indications and Timing

Once an irreducible anterior shoulder dislocation is suspected based on failed closed reduction attempts and confirmatory imaging, prompt surgical consultation is essential. Continued attempts at forceful manipulation risk further soft tissue injury, fractures, neurovascular compromise, and increased difficulty of subsequent open reduction [8]. Persistent deformity, pain despite adequate sedation, or radiographic evidence of incomplete reduction should trigger escalation to advanced imaging and surgical planning.

Surgical Approach

The deltopectoral approach is the most commonly utilized access for open reduction and has been employed in all contemporary cases [9-14,16]. This approach provides excellent visualization of the anterior glenohumeral joint, subscapularis, LHBT, anterior capsule, and neurovascular structures. The internervous plane is developed between the deltoid and pectoralis major, with the cephalic vein mobilized laterally. Key surgical steps include identifying and protecting neurovascular structures, exploring the anterior joint and identifying all interposed structures, gently reducing the humeral head after removing interposed tissue, repairing torn structures, and addressing associated fractures [33].

The role of arthroscopy is extremely limited. Connolly et al. attempted arthroscopy but converted to open reduction due to difficulty managing substantial soft tissue interposition [6]. Bayoumy et al. specifically advised that arthroscopy has no role when the joint space is occupied by the subscapularis tendon [9]. However, a systematic review by Liechti et al. identified one case managed arthroscopically among 12 cases, suggesting that arthroscopic techniques may be feasible in highly selected cases with minimal soft tissue interposition [12]. The overwhelming evidence supports open surgical approaches as the standard of care.

Intraoperative Techniques and Management of Specific Interposed Structures

Intraoperative external rotation of the shoulder joint is particularly helpful in delivering interposed tissue, especially when the subscapularis tendon is entrapped [9]. This technique was consistently utilized where subscapularis interposition was identified, helping to unfold and deliver the torn tendon stump from the joint space.

When the LHBT is interposed, management options include tenodesis, which involves reattachment to the proximal humerus and is preferred in younger active patients to prevent Popeye deformity and maintain biceps function, or tenotomy, which involves simple release without reattachment and is appropriate for elderly low-demand patients [34]. Associated greater tuberosity fractures require anatomical reduction and stable fixation using sutures, anchors, or screws, depending on the fragment size and bone quality [35].

Subscapularis interposition requires complete mobilization of the muscle-tendon unit, gentle extraction from the joint using external rotation maneuvers, and primary anatomical repair to the lesser tuberosity using suture anchors [36]. In elderly patients with poor tissue quality, augmentation with dermal allograft patches may be considered [37]. Lesser tuberosity fractures require anatomical reduction and stable fixation to restore the normal insertion site [11,15].

Management of massive rotator cuff tears with interposition requires thorough assessment of tissue quality, tear size, degree of retraction, and overall repairability. Treatment options include primary anatomical repair when tissue quality is adequate and mobilization achievable, partial repair when complete anatomical repair is not feasible, superior capsule reconstruction in younger patients with irreparable tears, or reverse total shoulder arthroplasty for elderly low-demand patients with irreparable cuffs and concurrent glenohumeral arthritis [38,39].

Nerve interposition, though exceptionally rare, requires meticulous dissection with magnification, gentle release of the entrapped nerve without applying traction, visual inspection for laceration or severe contusion, and possible neurosurgical consultation for primary repair if transection is identified [40]. Postoperative nerve conduction studies and electromyography at 3-6 weeks establish a baseline for monitoring recovery [41].

Rehabilitation

Rehabilitation protocols must balance tissue healing with early mobilization to prevent stiffness. Phase 1 (weeks 0-6) emphasizes tissue healing with sling immobilization for 3-6 weeks, depending on repair quality; passive range of motion exercises beginning at 2-3 weeks; immediate encouragement of active elbow, wrist, and hand exercises; and no active shoulder motion to protect repairs. Phase 2 (weeks 6-12) transitions to progressive active-assisted range of motion exercises, active range of motion, initiation of rotator cuff strengthening exercises, and scapular stabilization exercises. Phase 3 (3-6 months) focuses on progressive resistance training, sport-specific or occupation-specific exercises, and return to full activities as tolerated [42].

Outcomes and prognosis

The literature on outcomes consists primarily of case reports and small series with limited follow-up. The systematic review by Liechti et al. analyzing 12 cases reported no recurrent dislocations during available follow-up periods ranging from 6 weeks to 2 years [12]. Individual case reports generally describe favorable outcomes with restoration of shoulder function, although residual deficits are common. Pantazis et al. reported a Constant score of 90 at 12 months following open reduction and repair of LHBT interposition [10]. Sifi et al. achieved a Constant-Murley score of 85 at 12 months after managing the subscapularis and lesser tuberosity interposition; however, the patient demonstrated persistent limitation of internal rotation [15].

Factors associated with superior outcomes include early recognition and surgical intervention within 1-2 weeks of injury, younger patient age, absence of massive irreparable rotator cuff tears, successful anatomical reduction without recurrent instability, and compliance with rehabilitation protocols [43]. Poor prognostic factors include delayed presentation beyond 3-4 weeks, advanced patient age with poor tissue quality, massive irreparable rotator cuff tears, associated neurovascular injury, and significant articular cartilage damage with risk of post-traumatic arthritis development [44].

Complications

Complications associated with irreducible anterior shoulder dislocations and their surgical management are significant. Nerve injuries complicate 5%-55% of anterior shoulder dislocations, with higher rates in older patients and those with delayed presentation [26]. The axillary nerve is most commonly affected, resulting in deltoid paralysis and sensory loss over the lateral shoulder. Most nerve injuries represent neuropraxia from stretch or contusion and recover spontaneously over 3-6 months [27].

Vascular injuries are rare in young patients but increase significantly in patients over 50 years, particularly those with atherosclerotic disease [27]. Axillary artery injury can manifest as absent distal pulses, expanding hematoma, or acute ischemia requiring emergent vascular surgery consultation [45].

Post-traumatic arthritis is a significant long-term complication. Articular cartilage damage at the time of dislocation, combined with surgical manipulation and potential chondral injury during open reduction, increases the risk of progressive glenohumeral arthritis [46]. Patients may develop progressive pain, stiffness, and functional limitation over subsequent years.

Although properly reduced and surgically repaired irreducible dislocations have low rates of recurrent dislocation, residual instability symptoms, including subluxation and apprehension, can persist. Young, active patients with significant capsulolabral disruption face a higher risk of instability [47]. Stiffness and adhesive capsulitis risk increase with prolonged immobilization, particularly in older patients. Physical therapy plays a critical role in preventing and treating postoperative stiffness [48]. Rotator cuff dysfunction from irreparable tears, failed repairs, or progressive cuff degeneration can result in chronic weakness, pain, and functional limitation. Massive cuff tears may progress to rotator cuff arthropathy, requiring salvage procedures, including reverse total shoulder arthroplasty [49].

## Conclusions

Irreducible anterior shoulder dislocations caused by soft tissue interposition are rare but clinically significant, requiring prompt recognition and surgical management. The LHBT is the most frequently interposed structure, followed by the subscapularis tendon. Clinical suspicion should arise when closed reduction fails, despite adequate anesthesia and muscle relaxation. MRI is the gold standard for identifying interposed structures and planning surgical intervention, providing superior soft tissue characterization compared to CT. The deltopectoral approach remains the standard surgical access, with key technical principles including gentle tissue handling, use of external rotation maneuvers to deliver interposed subscapularis, anatomical reduction, secure repair of torn structures, and appropriate management of associated fractures. Outcomes are generally favorable, with low recurrence rates; however, complications, including nerve injury, post-traumatic arthritis, and residual functional deficits, can occur. Early recognition and intervention, meticulous surgical technique, and structured rehabilitation protocols are essential to optimize patient outcomes.
